# Effect of different habitat types on abundance and biting times of *Anopheles balabacensis* Baisas (Diptera: Culicidae) in Kudat district of Sabah, Malaysia

**DOI:** 10.1186/s13071-019-3627-0

**Published:** 2019-07-25

**Authors:** Tock H. Chua, Benny O. Manin, Indra Vythilingam, Kimberly Fornace, Chris J. Drakeley

**Affiliations:** 1 Department of Pathobiology and Medical Diagnostics, Faculty of Medicine and Health Sciences, Universiti Malaysia Sabah, Kota Kinabalu, Sabah, Malaysia; 2 Department of Parasitology, Faculty of Medicine, University of Malaya, Kuala Lumpur, Malaysia; 3 Faculty of Infectious and Tropical Diseases, London School of Hygiene and Tropical Medicine, London, UK

**Keywords:** *Anopheles balabacensis*, Abundance, Habitat type, Biting time, Malaria vector, *Plasmodium knowlesi*, Sabah, Malaysia

## Abstract

**Background:**

We investigated the effect of five common habitat types on the diversity and abundance of *Anopheles* spp. and on the biting rate and time of *Anopheles balabacensis* (currently the only known vector for *Plasmodium knowlesi* in Sabah) at Paradason village, Kudat, Sabah. The habitats were forest edge, playground area, longhouse, oil palm plantation and shrub-bushes area. Sampling of *Anopheles* was done monthly using the human landing catch method in all habitat types for 14 months (October 2013 to December 2014, excluding June 2014). The *Anopheles* species were morphologically identified and subjected to PCR assay for the detection of *Plasmodium* parasites. Generalised linear mixed models (GLMM) were applied to test the variation in abundance and biting rates of *An. balabacensis* in different habitat types.

**Results:**

A total of 1599 *Anopheles* specimens were collected in the village, of which about 90% were *An. balabacensis*. *Anopheles balabacensis* was present throughout the year and was the dominant *Anopheles* species in all habitat types. The shrub bushes habitat had the highest *Anopheles* species diversity while forest edge had the greatest number of *Anopheles* individuals caught. GLMM analysis indicated that *An. balabacensis* abundance was not affected by the type of habitats, and it was more active during the early and late night compared to predawn and dawn. PCR assay showed that 1.61% of the tested *An. balabacensis* were positive for malaria parasites, most of which were caught in oil palm estates and infected with one to two *Plasmodium* species.

**Conclusions:**

The identification of infected vectors in a range of habitats, including agricultural and farming areas, illustrates the potential for humans to be exposed to *P. knowlesi* outside forested areas. This finding contributes to a growing body of evidence implicating environmental changes due to deforestation, expansion of agricultural and farming areas, and development of human settlements near to forest fringes in the emergence of *P. knowlesi* in Sabah.

**Electronic supplementary material:**

The online version of this article (10.1186/s13071-019-3627-0) contains supplementary material, which is available to authorized users.

## Background

Borneo Island, the third largest island in the world, is famous for its diverse and unique flora and fauna. Unfortunately, Borneo Island has been experiencing forest losses recently. From 1975 to 2015, approximately 18.5 million hectares (Mha) of old forests were cleared for industrial plantations [1]. In 2015, an estimated 7.9 Mha of the total Borneo landmass (73.7 Mha) were planted with oil palm. Changes in the environment due to deforestation, mainly for development, logging and agricultural plantations, are also changing the ecology of animals on the island, including anopheline ecology [2]. With continued deforestation across Borneo [3], resulting fragmentation of forest areas may increase the spillover risk of zoonotic, vector-borne diseases due to increased contact between humans and disease reservoirs and vectors [4].

Sabah, the second largest state in Malaysia, situated in northern Borneo Island with an estimated area of 73,620 km^2^ has recently experienced tremendous forest loss. Prior to the 1980s, about 80% of Sabah was covered with mainly dipterocarp forests [5]. However, by the mid-1980s, only 60% of Sabah was covered with forest, a loss of 20% in a short time due to logging and agriculture activities. By 1995, about 90% of the primary forests in Sabah had been lost, with the area reduced from around 2.8 to 0.3 Mha [5].

One of the current major mosquito borne-diseases in Sabah is zoonotic knowlesi malaria caused by *Plasmodium knowlesi* transmitted from long-tailed and pig-tailed macaques by *Anopheles* mosquitoes [6]. In 2016, 93% of the knowlesi malaria cases in Malaysia (1263/1357) were reported from Malaysian Borneo [7]. Deforestation and land alteration have been thought as the main reasons behind the increase of knowlesi malaria cases by creating new habitat types suitable for the *Anopheles* vector [8].

In Southeast Asia, *Anopheles* species are present in high diversity, including species complexes that occur in sympatry but have different behavior [9, 10]. The high diversity of these vectors and the complexity of the species could lead to misidentification which may influence the success of vector control programmes [11, 12]. In East Malaysia, *An. balabacensis* and *An. latens* have been confirmed as the main vectors for *P. knowlesi* in Sabah and Sarawak, respectively [13, 14]. In Peninsular Malaysia, several species of *Anopheles* belonging to the Leucosphyrus group had been identified as the natural vectors for *P. knowlesi* [15]. Nevertheless, other *Anopheles* sp. (e.g. *An. kochi*) can act as vectors for *P. knowlesi* but further studies are needed to establish their role in transmission [16].

In Asia, *P. knowlesi* was probably the first simian malaria confirmed to cause widespread human infection after a group of villagers had been found infected naturally in Kapit, Sarawak, East Malaysia in 2004 [17]. This species was previously misidentified as *P. falciparum* or *P. malariae* [18] until molecular techniques were introduced. Since then, *P. knowlesi* cases have also been reported in Sabah [19–21] and Peninsular Malaysia [22–24]. Similarly, many other countries in Southeast Asia have also reported cases, e.g. Kalimantan, Indonesia [25, 26]; Sumatra, Indonesia [27]; Brunei [28]; Thailand [29, 30]; Vietnam [31, 32]; Singapore [16, 33]; Phillippines [34]; Myanmar [35]; Cambodia [36]; and recently Laos [37].

In this study, we conducted an entomological investigation to determine the diversity and abundance of *Anopheles* species, especially *An. balabacensis*, in the five types of habitats that are now commonly found in the rural areas of Sabah. *Anopheles balabacensis* has been known as the dominant species in Kudat Division, mainly in Banggi Island located off the northern coast of Sabah near Marudu Bay [38]. Current changes in the landscape of Kudat district of Kudat Division have provided new habitats for *An. balabacensis* closer to human settlements, have altered its distribution, increased their opportunity of feeding on humans and infecting them with malaria parasites.

## Methods

### Sampling site

Kudat Division of Sabah is the smallest division, and covers the districts of Kudat, Pitas, Kota Marudu, Balambangan, Banggi Island and several other islands. The climate in Sabah is tropical with an average temperature of 32 °C for lowland areas and 21 °C for highland areas, typically hot and sunny the whole year. Rainfall is common throughout the year and influenced by northeast (November to March) and southwest monsoons (May to September).

**Fig. 1:**
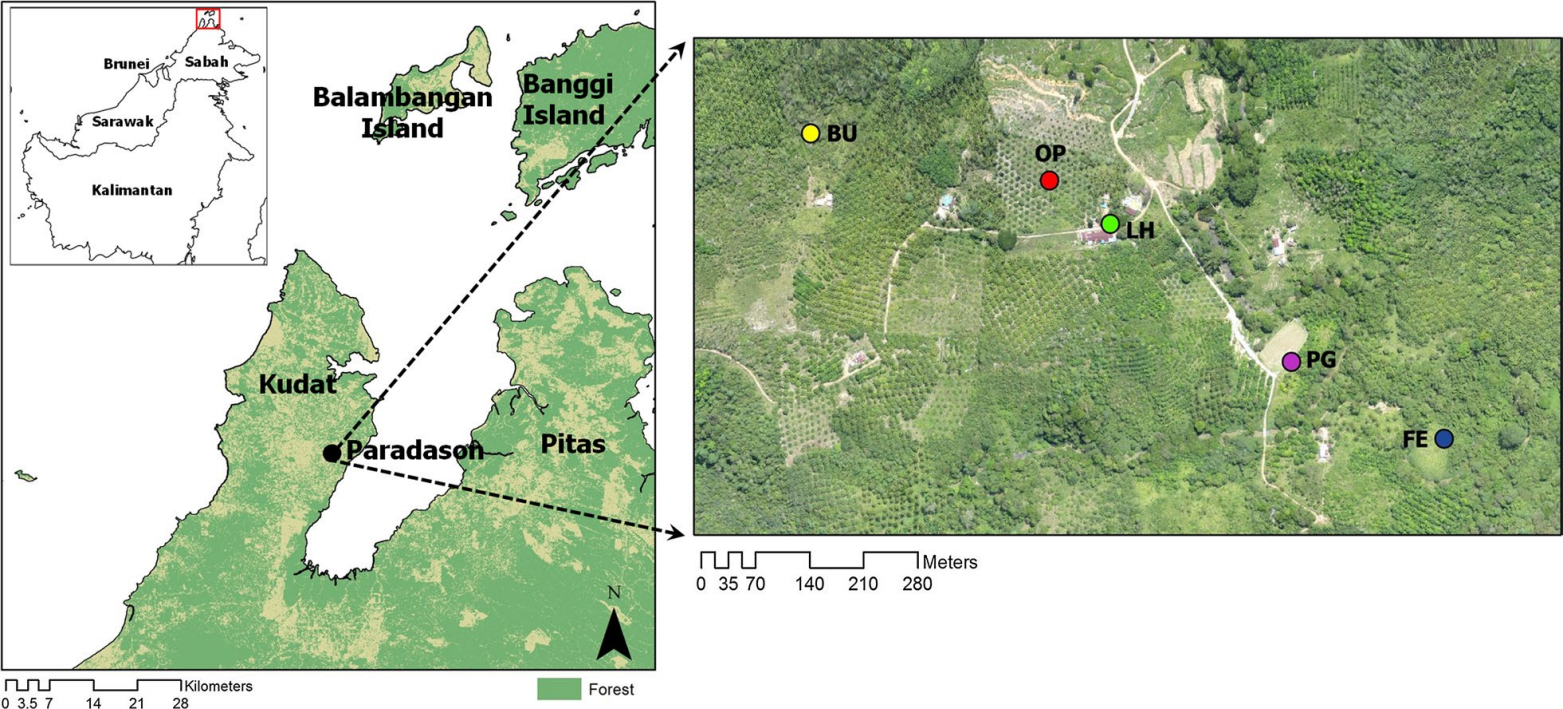
Map showing the location of Kudat district, location of Paradson village and the five types of habitat (or sampling sites) labeled as FE, PG, LH, OP and BU. *Abbreviations*: FE, forest edge between forest and deforested area; PG, playground area surrounded with small trees including bamboo, coconut and rubber trees; LH, area outside a longhouse; OP, an oil palm plantation; BU, shrub-bushes area with small and tall grasses near to a coconut plantation

This study was carried out in Kudat district (Fig. 1) which receives a high volume of rain during the northeast monsoon but less rain during the southwest monsoon. The population of Kudat district was about 85,300 in July 2010 with the majority of Rungus ethnicity [39]. They live in several hamlets each consisting of several houses or longhouses. The villagers practice swidden farming, growing rice, corn and vegetables for their own consumption. Some families might have their own small plantation of coconut, rubber or oil palm but many work as laborers in oil palm plantations.

**Fig. 2:**
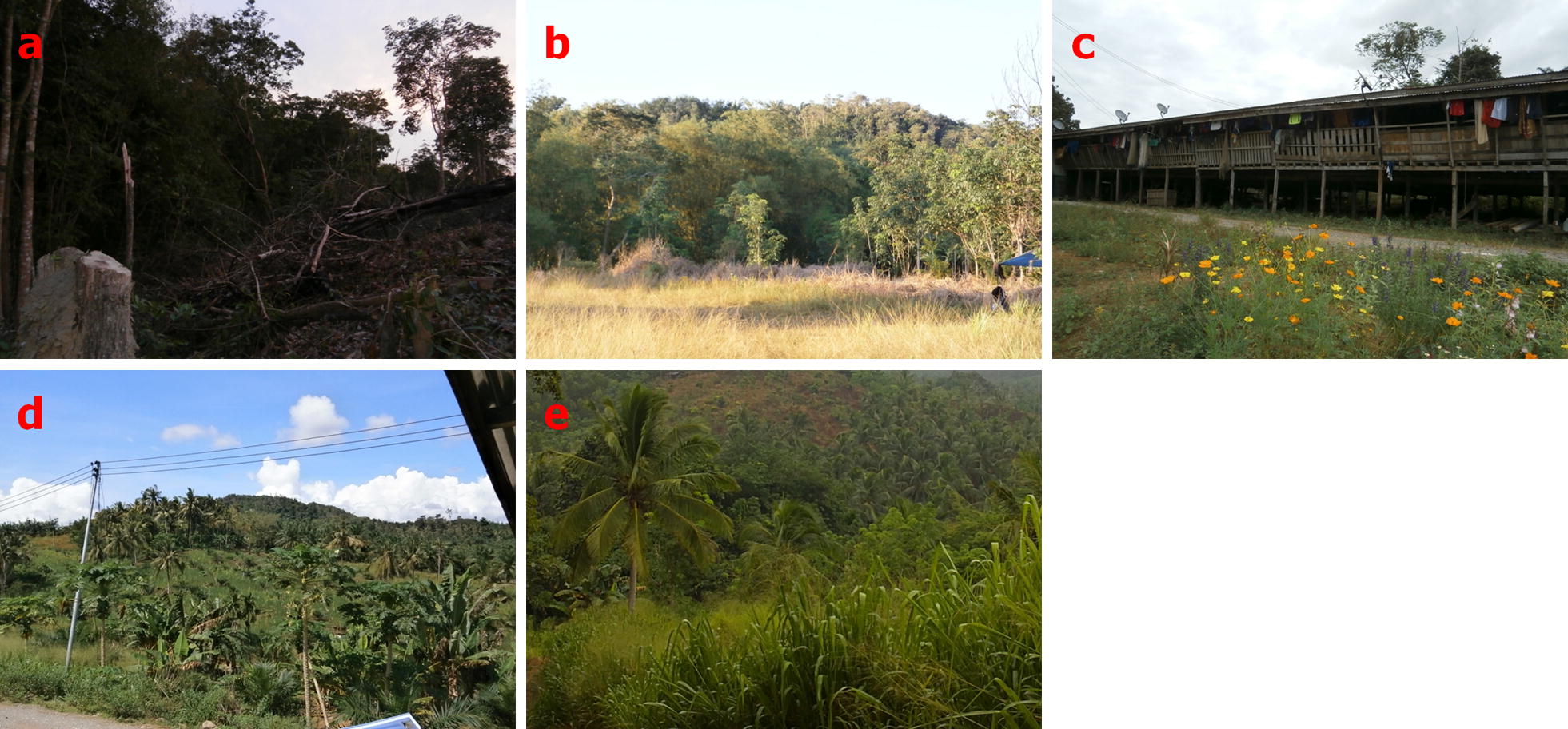
The habitat types where mosquitoes were sampled are: **a** FE, forest edge between forest and deforested area; **b** PG, playground area surrounded with small trees including bamboo, coconut and rubber trees; **c** LH, area outside a longhouse; **d** OP, an oil palm plantation; and **e** BU, shrub-bushes area with small and tall grasses near to a coconut plantation

Sampling of *Anopheles* mosquitoes was conducted in Paradason village [40] (6°46′11.75″N, 116°47′3.23″E) which had 28 households and 122 residents in October 2013 census (Fig. 1). Prior to and within the study period five knowlesi malaria cases had been reported from this village. Long-tailed macaques were also sighted in the surrounding areas. The village has an area of about 1.98 km^2^ comprising forest, plantation, farm and human settlement areas. Five types of habitats were selected: a forest edge between secondary forest and deforested area (FE); a playground area (or an open space area) surrounded with small trees including bamboo, coconut and rubber trees where the village youths gather during evening to play football (PG); an area outside a longhouse (LH); a farm which was converted into an oil palm plantation (OP); and a shrub-bushes area with small and tall grasses near to a coconut plantation (BU) (Fig. 2).

*Anopheles* mosquitoes were collected by the human landing catch (HLC) method, with two persons in a team, which involved exposing lower part of their legs to attract mosquitoes. Mosquitoes that landed on their legs were captured using a plastic specimen tube (2 cm diameter × 6 cm length). Each captured mosquito was placed in a separate specimen tube which was labeled with the time of capture. The mosquitoes were sampled from 18:00–06:00 h (12 h). Sampling was done once a month for 14 months starting in October 2013 and ending in December 2014 with no sampling in June 2014. Meteorological data such as temperature, humidity and rainfall were obtained from Kudat meteorological station which was located 17 km from Paradason village.

### Morphological identification of *Anopheles* mosquitoes

The *Anopheles* mosquitoes were killed by keeping them in a freezer (− 20 °C) and each individual was identified based on morphological characteristics using published keys [41–44]. *Anopheles* specimens were mounted onto Nu poly strip using ultra-thin micro-headless pin and identified under a compound microscope. Each individual mosquito was kept separately in a clean microfuge tube and transported to the Faculty of Medicine & Health Sciences, Universiti Malaysia, Sabah, for further processing.

### Species composition, diversity and evenness

The diversity index of *Anopheles* mosquitoes in each habitat type was determined using the Shannon–Wiener index, $H$ [45]. This index is commonly used to characterize species diversity in a community which accounts for both abundance and evenness of the species present. Evenness assumes a value between 0 and 1 with 1 being complete evenness. The index is calculated based on the formula, $H = - \mathop \sum \nolimits_{i = 1}^{S} P_{i} \left( {{\text{ln }}P_{i} } \right)$ where $S$ is the number of species and $P_{\text{i}}$ is the proportion of individuals of i_*th*_ species.

### Extraction of total DNA from *Anopheles* mosquitoes

Each *Anopheles* individual was placed separately in a clean mortar and the tissue was homogenized using a sterile pestle. The total DNA was extracted from each individual using the DTAB-CTAB method [46] with some modifications. First, 600 µl of DTAB solution was added into the mortar and the tissue was ground using pestle until homogenized. Then, the homogenized tissue was transferred into a clean 1.5 ml microcentrifuge tube and incubated at 68 °C for 30 min. Subsequently, 700 µl of chloroform was added into the microcentrifuge tube which was inverted ten times to mix the contents and centrifuged at 13,000× *rpm* for 5 min. Then, 400 µl of the upper aqueous layer was carefully transferred into a new clean 1.5 ml microcentrifuge tube and mixed with 900 µl of sterile dH_2_O and 100 µl CTAB solution by gently inverting the microcentrifuge tube several times. The tube was then allowed it to sit at room temperature for 5 min and spun at 13,000× *rpm* for 10 min. The supernatant was discarded and the DNA pellet was re-suspended in 300 µl of 1.2 M NaCl solution. Total DNA was precipitated by adding 750 µl of absolute ethanol and centrifuged at 13,000× *rpm* for 5 min. Then, the supernatant was discarded and the DNA was washed with 500 µl of 70% ethanol and centrifuged at 13,000× *rpm* for 2 min. The DNA pellet was air-dried at 45 °C for 10 min, re-suspended in 30 µl of Tris-EDTA (pH8.0) buffer and stored at − 30 °C until use.

### Detection of malaria parasites

The presence of malaria parasites in the mosquitoes was detected using nested PCR assay by targeting the small subunit ribosomal RNA (*SSU* rRNA) gene of *Plasmodium*. The PCR primer pair rPLU1 and rPLU5 were used in first PCR reaction, while rPLU3 and rPLU4 were used in the second reaction [47]. For internal control, another nested PCR reaction was performed separately at the same time to amplify the cytochrome *c* oxidase subunit 2 (*cox*2) gene of *Anopheles* [48]. When a specimen was found positive for malaria parasites, further identification of *Plasmodium* sp. was conducted using nine species-specific primers (Additional file 1: Table S1). For positive control, known positive DNA of each *Plasmodium* species was obtained from the Parasitology and Medical Entomology Laboratory, Faculty of Medicine and Health Sciences, Universiti Malaysia Sabah and tested with the nine species-specific PCR primers (Additional file 2: Figure S1).

Both PCR genus-specific and species-specific reactions were performed in a final volume of 25.0 µl. Reaction components were prepared by mixing 5.0 µl of 5× PCR buffer (Promega, Madison, WI, USA), 0.5 µl of (10 mM) dNTPs (Promega), 3.0 µl of (25 mM) MgCl_2_, 1.0 µl of (10 µM) forward and reverse primers, 0.3 µl of (5.0 U/µl) Taq DNA polymerase (Promega), 2.0 µl of DNA template and sterile dH_2_O up to 25.0 μl final volume. After completion of the first PCR, 2.0 µl of the PCR products was used as a DNA template in the second PCR. The reaction was carried out using a thermal cycler (T100™ Thermal Cycler, Bio-Rad, California, CA, USA) with the following conditions: initial denaturation at 95 °C for 5 min; 35 cycles of denaturation at 94 °C for 1 min, annealing for 1 min and extension at 72 °C for 1 min; final extension at 72 °C for 5 min. The annealing temperature used was based on the optimal temperature for each set of primers (Additional file 1: Table S1). PCR products were analyzed on 1.5% agarose gel electrophoresis stained with RedSafe™ nucleic acid staining solution (iNtRON Biotechnology, Jungwon-Seongnam, South Korea) and visualized using UV transilluminator.

### Analysis of relationship between *An. balabacensis* and meteorological data

The correlation between the number of *An. balabacensis* caught per person per night with temperature, relative humidity and rainfall from Kudat meteorological station was examined using Pearson’s linear correlation coefficient (*r*) and checked for significance. The test was conducted using SPSS v.18 for Windows 10.

### Analysis of abundance of *An. balabacensis*

Statistical analysis of mosquito abundance was conducted using R programming software for statistical analysis (v.3.2.2). Generalised linear mixed models (GLMM) were constructed to test the variation in abundance of *An. balabacensis* related to month of collection, types of habitat and time of biting. This model included both fixed and random predictor variables in the analysis. Time of biting was categorized into four periods for the total catch of *An. balabacensis*: 18:00–21:00 h (early night); 21:00–00:00 h (late night); 0:00–03:00 h (predawn); and 03:00–06:00 h (dawn). In the analysis, the type of habitat and the time of biting were considered as fixed effects. Month was fitted alternatively as fixed (to predict monthly value) and random effect (to test differences between types of habitat and time of biting while controlling variation in seasonality). In order to determine the best model, both negative binomial and Poisson distributions including zero inflation were tested in the model. Tukey’s *post-hoc* test was used to compare predicted means between the fixed effects and the interaction between the two fixed effects.

## Results

### *Anopheles* species composition, diversity and evenness

A total of ten *Anopheles* species were recorded from Paradason village (Table 1). The highest number of species was recorded from shrub bushes (BU, 10 species) and the least was from oil palm plantation (OP, 4 species). *Anopheles balabacensis* was the dominant species in all types of habitat with the total number caught reaching 90% (1437/1599). The highest number of *Anopheles* was recorded from the forest edge (FE, 21.58%) followed by oil palm (OP, 20.39%) and shrub bushes (BU, 20.39%), longhouse (LH, 19.07%) and playground (PG, 18.57%). Overall, the diversity of *Anopheles* mosquito in Paradason village is considered low as indicated by the evenness value of 0.2.

**Table 1:** Total numbers of *Anopheles* species caught from each type of habitat from October 2013 to December 2014

Species	FE	PG	LH	OP	BU	Total
*An. balabacensis*	316	264	274	299	284	1437
*An. barbumbrosus*	13	14	20	21	24	92
*An. donaldi*	9	4	2	0	4	19
*An. maculatus*	2	10	5	0	5	22
*An. nigerrimus*	3	0	1	0	1	5
*An. peditaeniatus*	0	3	0	0	1	4
*An. separatus*	0	0	0	0	1	1
*An. sundaicus*	0	0	1	0	1	2
*An. tessellatus*	2	2	2	4	4	14
*An. umbrosus*	0	0	0	2	1	3
Total no. of specimens	345	297	305	326	326	1599
Total no. of species	6	6	7	4	10	10
Shannon–Wiener index, $H$	0.40	0.50	0.45	0.34	0.57	0.47
$H$_max_	1.79	1.79	1.95	1.39	2.30	2.30
Evenness	0.22	0.28	0.23	0.25	0.25	0.20

*Abbreviations*: FE, forest edge; PG, playground; LH, longhouse; OP, oil palm plantation; BU, shrub bushes

### Abundance of *An. balabacensis* by month and habitat type

**Fig. 3:**
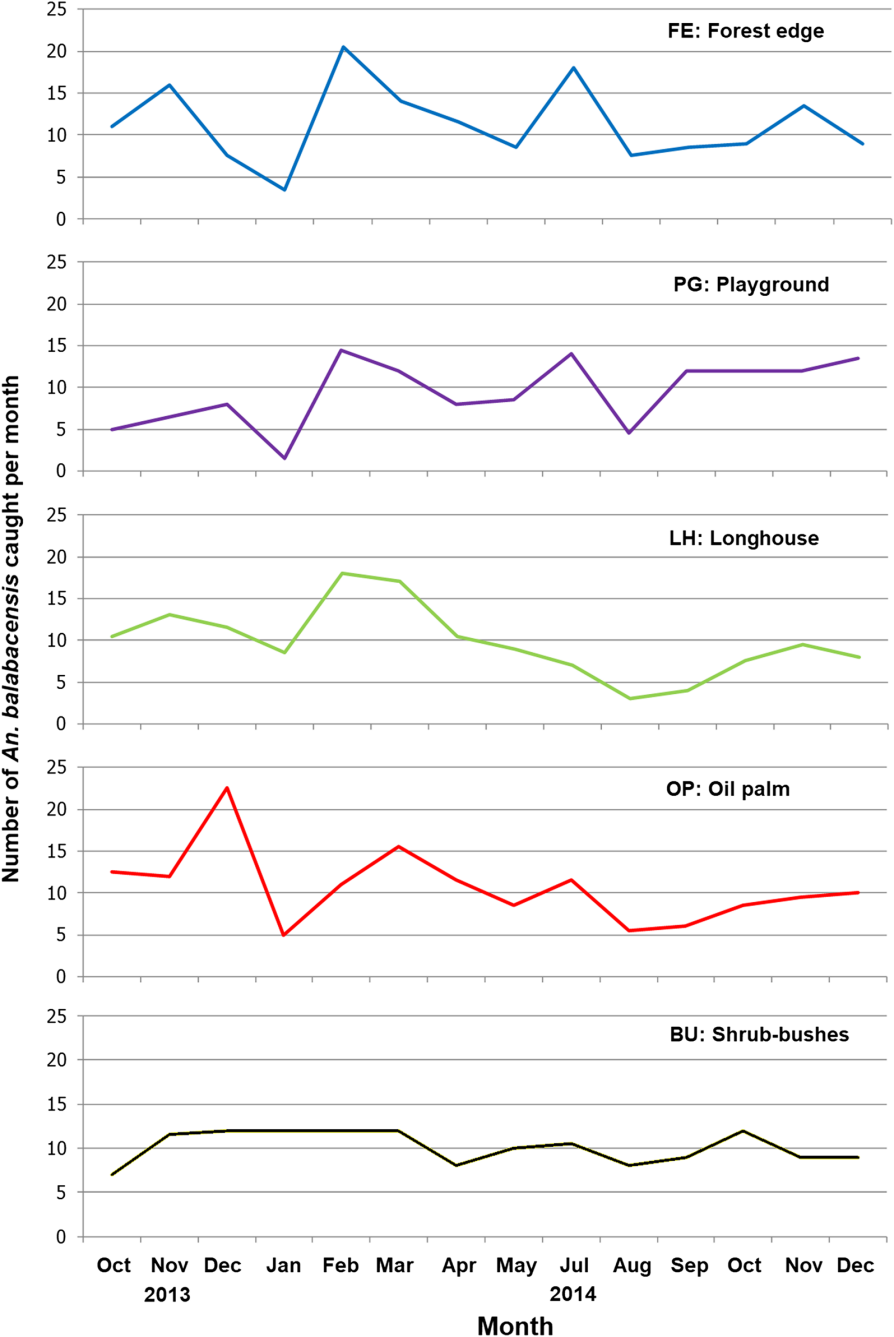
Number of *An. balabacensis* caught in the five types of habitat in each month from October 2013 to December 2014. FE, forest edge between forest and deforested area; PG, playground area surrounded with small trees including bamboo, coconut and rubber trees; LH, area outside a longhouse; OP, an oil palm plantation; and BU, shrub-bushes area with small and tall grasses near to a coconut plantation

A total of 1437 individuals of *An. balabacensis* were caught for the 70 sampling nights pooling all the habitats at Paradason village, equivalent to 10.3 ± 0.47 bites per man per night. The fluctuations in the monthly number of *An. balabacensis* caught in the five types of habitat showed similar trends (Fig. 3). The GLMM predicted the highest mean monthly number of *An. balabacensis* for February (15.0 ± 3.06 bites per man per night) and the lowest for August (5.40 ± 1.52 bites per man per night). Fitting the model with a Poisson distribution and comparing the predicted mean between all months showed that there was a significant difference only between December 2013 and August 2014 (12.0 ± 1.55 bites per man per night *versus* 5.40 ± 1.52 bites per man per night, *Z* = 3.44, *P* = 0.0372) and between February 2014 and August 2014 (15.0 ± 3.06 bites per man per night *versus* 5.40 ± 1.52 bites per man per night, *Z* = − 3.59, *P* = 0.0231).

The mean bites per man per night for each habitat type predicted by GLMM were 10.7 ± 1.67, 10.1 ± 1.58, 9.8 ± 1.03, 9.3 ± 1.50 and 9.0 ± 1.50 for forest edge, oil palm estates, bushes, longhouse and playground, respectively. However, using a Poisson distribution in the model, no difference was detected in the number caught between the different habitat types.

**Fig. 4:**
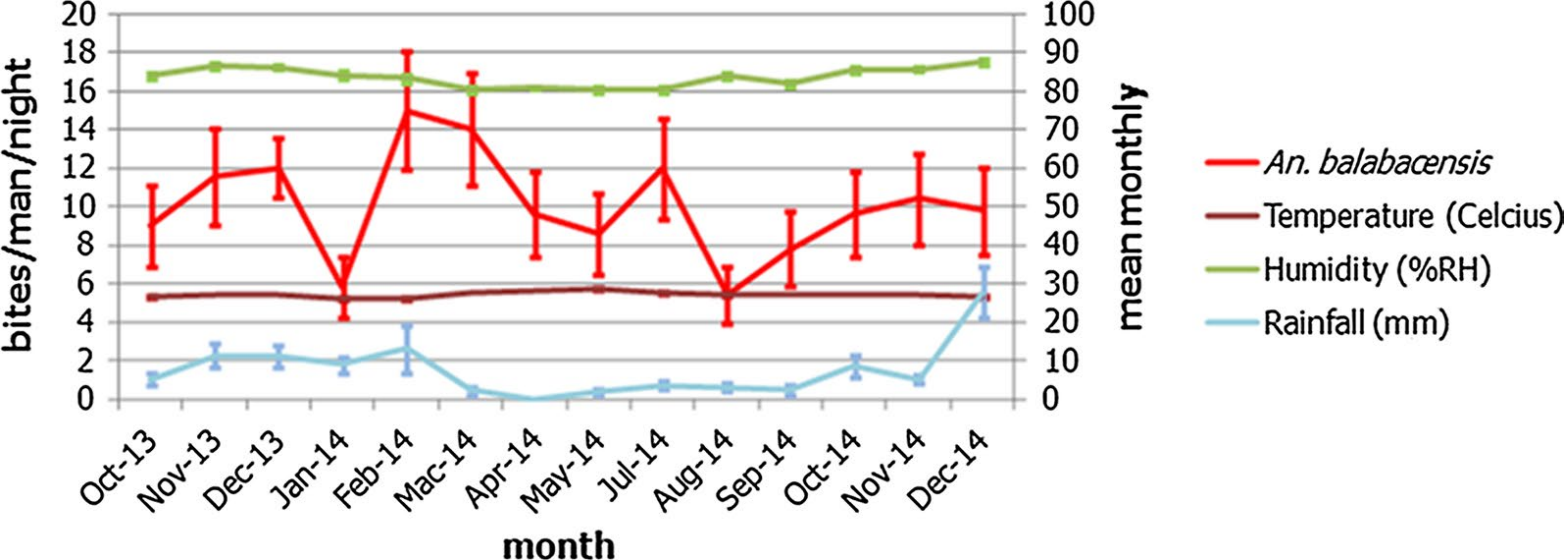
Monthly mean temperature, relative humidity and rainfall and the number of *An. balabacensis* collected in Kudat district from October 2013 to December 2014

**Fig. 5:**
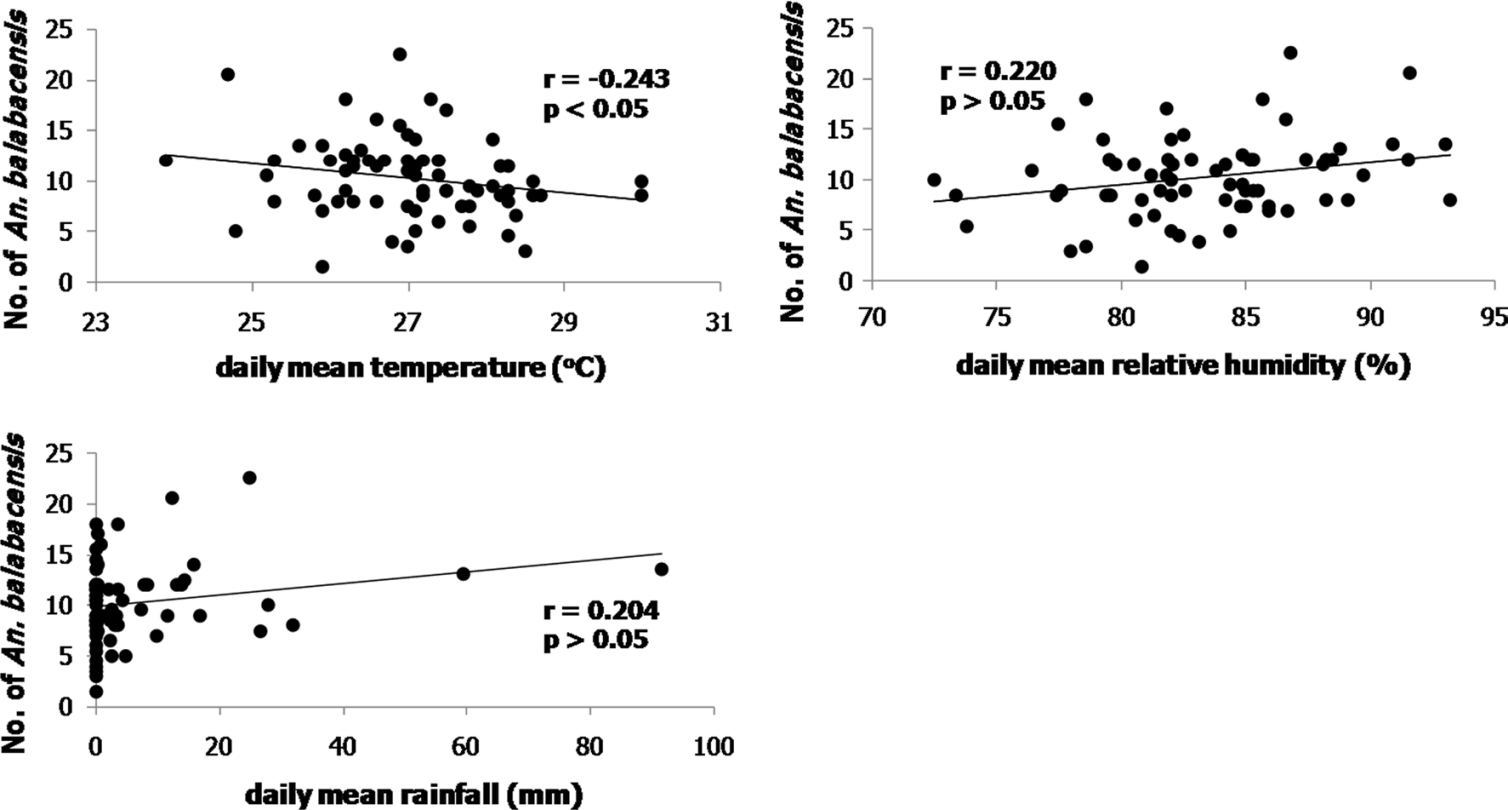
Plot of *An. balabacensis* (bites/man/night) caught on each sampling night (*n* = 70) against the environmental factors: mean daily temperature; mean daily relative humidity; and mean daily rainfall

During the study period, the range of the mean monthly temperature, relative humidity and rainfall in Kudat district were 25.9–28.4 °C, 80.4–87.5% RH and 0–27.8 mm, respectively (Fig. 4). Analysis of the number of *An. balabacensis* caught on each sampling night against the environmental factors of the day showed significant correlation between the numbers of *An. balabacensis* caught with temperature (*r* = − 0.243, *P* = 0.043), but not with relative humidity (*r* = 0.220, *P* = 0.067) and rainfall (*r* = 0.204, *P* = 0.090) (Fig. 5).

### Biting time of *An. balabacensis*

**Fig. 6:**
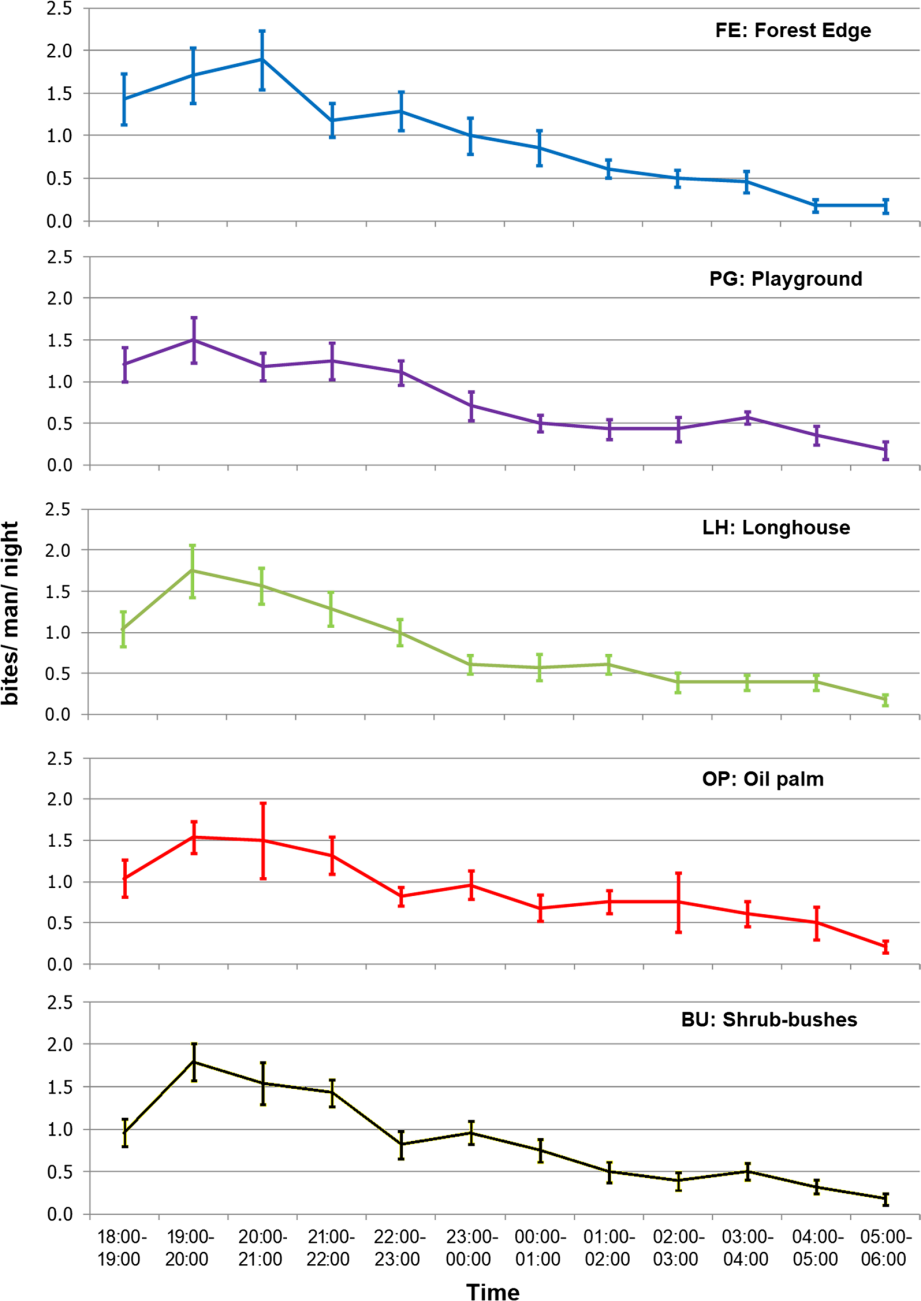
Mean number of bites/man/night (and 95% standard error bars) of *An. balabacensis* recorded in each hour for the various types of habitat

Overall, biting times of *An. balabacensis* from different types of habitat was similar with a peak biting time of 19:00–20:00 h in PG, LH, OP and BU and 20:00–21:00 h in FE (Fig. 6). More *An. balabacensis* were caught within the first three hours after dusk (18:00–21:00 h) with the number decreasing gradually until dawn. Only one mosquito bite per person or less in each hour after midnight was recorded. Fitting the model with a Poisson distribution, there were significant differences between the catch at early night *versus* predawn, early night *versus* dawn, late night *versus* predawn, late night *versus* dawn, and predawn *versus* dawn (Table 2).

**Table 2:** Mean biting rate for each time period of catch as predicted by GLMM

Time period	Time	Mean ± SE (bites/man/time period)	Significant differences^a^
Early night	18:00–21:00 h	3.9 ± 0.72	Early night *vs* Late night: *P* = 0.2472; Early night *vs* Predawn: *P *< 0.0001;
Late night	21:00–00:00 h	2.8 ± 0.53	Early night *vs* Dawn: *P *< 0.0001; Late night *vs* Predawn: *P* = 0.0024;
Predawn	00:00–03:00 h	1.4 ± 0.29	Late night *vs* Dawn: *P *< 0.0001; Predawn *vs* Dawn: *P* = 0.0011
Dawn	03:00–06:00 h	0.7 ± 0.11	

^a^Detected by Tukey’s test between the pairs of time periods

Analysis of interaction between habitat type and time-period showed that only three interactions were significant, namely FE × early night *versus* FE × dawn, BU × early night *versus* BU × dawn, and BU × late night *versus* BU× dawn (Additional file 3: Table S2).

### Presence of malaria parasites in *Anopheles* specimens

A total of 1586 *Anopheles* specimens were tested for malaria parasites using a PCR assay, and only 23 (1.45%) were found positive. These were all *An. balabacensis*, constituting 1.6% or 23/1425 of the species collected. Most infected specimens were caught in the oil palm plantation and shrub bushes, and during early and late night. These *An. balabacensis* were infected with one or two simian malaria species with *P. inui* being the most common species, while *P. knowlesi* was found in two mosquitoes only (Table 3; representative figures of agarose gels are shown in Additional file 4: Figure S2).

**Table 3:** Number of *Plasmodium-*infected *An. balabacensis* caught from each habitat type, the recorded biting time and the *Plasmodium* species as detected using PCR assay

Habitat	No. of infected *An. balabacensis*	Biting time	*Plasmodium* spp.
Forest edge (FE) (*n* = 315)	4	Early night	*P. knowlesi* + *P. cynomolgi*
		Late night (*n* = 2)	*P. inui*, *P. cynomolgi*
		Predawn	*P. inui*
Playground (PG) (*n* = 260)	1	Early night	*P. inui*
Longhouse (LH) (*n* = 270)	4	Late night	*P. inui*
		Predawn (*n* = 2)	*P. inui*, *P. cynomolgi*
		Dawn	*P. coatneyi*
Oil palm plantation (OP) (*n* = 299)	7	Early night (*n* = 4)	*P. inui* (*n* = 3), *P. coatneyi*
		Late night (*n* = 2)	*P. inui*, *P. fieldi* + *P. cynomolgi*
		Dawn	*P. inui*
Shrub bushes (BU) (*n* = 281)	7	Early night (*n* = 2)	*P. inui*, *P. inui* + *P. cynomolgi*
		Late night (*n* = 2)	*P. coatneyi*, *P. knowlesi* + *P. inui*
		Predawn (*n* = 2)	*P. cynomolgi* (*n* = 2)
		Dawn	*P. inui* + *P. cynomolgi*

*Abbreviation*: n, number of specimens

## Discussion

We conducted a survey at Paradason village, in Kudat district, Sabah to investigate if the diversity and abundance of adult *Anopheles* is influenced by the habitat types that are generally found in the rural areas of Sabah, namely forest edge, playground, longhouse, oil palm plantation and shrub bushes.

We collected ten *Anopheles* species from all the habitats, among which *An. balabacensis* was the dominant species in all types of habitat. The dominance of this species in Kudat Division was also noted previously [14], although our study in Tajau Laut village located further north of Kudat indicated that *An. balabacensis* and *An. tessellatus* were almost equally common [48]. However, differences in the dominant *Anopheles* species have been reported in different locations across the wider state of Sabah, possibly due to heterogeneities in the surrounding environments. For example, in the northern area of Ranau district, *An. maculatus* was recorded as the dominant species, followed by *An. balabacensis* and *An. donaldi* [49], while in Kinabatangan district *An. donaldi* was the dominant species replacing *An. balabacensis*, possibly due to the development and malaria control activities conducted there [50]. Our recent data from Paus village in southern part of Ranau district showed that the dominant species is *An. donaldi* followed by *An. balabacensis* and *An. barbumbrosus* (unpublished data). Further south in Keningau district, in Keritan Ulu village, *An. maculatus*, *An. balabacensis* and *An. barbumbrosus* were almost equally abundant (Additional file 5: Table S3). Overall, the diversity of *Anopheles* species in Paradason village is considered low as indicated by the evenness index due to overwhelming abundance of *An. balabacensis* in all types of habitat.

*Anopheles balabacensis* has been known as the dominant and common *Anopheles* species in Kudat Division of Sabah since 1980s [38] and as an efficient human malaria vector in Sabah since 1950s [51, 52]. Now, almost 40 years later, this species still ranks high among the dominant *Anopheles* species in Kudat district despite significant land use and land cover changes, due to development for human settlements, infrastructure, farming and plantations [8, 53]. Our findings suggest that *An. balabacensis* is adaptable and able to thrive in the man-made environment, with relatively high densities in plantation and farm areas. This is evident by the presence of *An. balabacensis* breeding sites observed in oil palm plantation near to human settlements. Similar inference was drawn for *An. cracens* in Peninsular Malaysia [54] and *An. dirus* (*s.l*.) in Thailand [55], both of which have adapted and colonized the forest edge and plantations after deforestation.

The shrub-bush habitat recorded the highest number of *Anopheles* species. This could be a result of shrub and bushes providing ample refuge areas for resting before and after blood meals [56], and shade for the breeding sites in both permanent and temporary water bodies. In the Paradason area, this bush habitat was observed in close proximity to human settlements, farms, plantations and deforested areas, which could be a source of infective *Anopheles*. Furthermore, a troop of long-tailed macaques was also observed in study site especially in the morning (06:00–07:00 h) and evening (16:00–18:00 h) coming from a nearby forest area into a coconut farm to forage for food. These monkeys were positive for *P. knowlesi* [57], just as those in other parts of Malaysia which recorded 50–97% positive [58–60].

Although forest activities have previously been associated with increased human risk of *P. knowlesi* within this area [61], GLMM analysis indicated that there was no difference in number of *An. balabacensis* caught at various habitat types, indicating individuals have a risk of being exposed to an infected mosquito across habitat types. Although the HLC was conducted on different nights at each habitat type which may introduce bias between sampling, *An. balabacensis* is known to have a short flight distance with an average maximum flight distance of less than 500 meters [62]. This suggests *An. balabacensis* is likely to stay in close proximity to breeding sites, as observed with *An. farauti* (*s.l.*) [10] and conducting HLCs on different nights between habitat types is not likely to substantially impact results of this study.

Data on the biting times indicate that *An. balabacensis* starts to be actively biting as soon as it gets dark, especially at the forest edge or bush areas that have been deforested, highlighting the need for vector control measures targeting these times when people are unlikely to be sleeping under a bednet. A previous study [63] also showed more *An. balabacensis* bite humans in logged forest as compared to primary and virgin reserve forests. This study did not identify a significant difference in abundance of *An. balabacensis* between early and late nights; however, human exposure may be higher earlier in the night when people are more likely to visit these environments. A previous study in Kudat demonstrated the risk of getting bitten by *An. balabacensis* was higher for those who stay outside the house after dusk (18:00–00:00 h) as compared to those who stay inside [64]. The number of *Anopheles* mosquitoes seeking blood meals at night might be dependent on natural preferences, host availability and environmental conditions such as temperature, relative humidity, rainfall, moonlight and wind speed [65–67]. *Anopheles balabacensis* in this study area showed a significant negative correlation with temperature but did not show a significant correlation with the increment of relative humidity and rainfall. The lack of correlation between the mosquito catch and rainfall could be due to the localized variations in rainfall and microclimate as data was only available from the Kudat meteorological station, located 17 kilometers from the sampling site at Paradason village.

Only 1.45% of all *Anopheles* specimens were found infected with simian *Plasmodium* and of these only *An. balabacensis* was positive. However, since whole mosquitoes were processed, infectious (oocyst positive) and infective (salivary glands positive) individuals cannot be differentiated. Most of the infections were by single species, *P. inui* (47.8%), followed by *P. cynomolgi* (17.4%). In addition, the two mosquitoes infected with *P. knowlesi* were also co-infected either with *P. inui* or *P. cynomolgi*. These two specimens were caught at the forest edge and shrub bushes habitats, respectively, where a troop of long-tailed macaques had been observed.

*Anopheles balabacensis* is a vector for *P. knowlesi* as well as for other simian malaria parasites [14, 64]. Furthermore, we could not detect any human malaria parasites by PCR in the *Anopheles* specimens although *P. falciparum* and *P. vivax* cases have been reported in Kudat district [19, 68]. This contrasts with findings in Vietnam in which *An. dirus* (*s.l*.), a primary vector for *P. knowlesi*, had co-infections with *P. vivax* and *P. falciparum* [31, 69]. The majority of the positive *An. balabacensis* was infected with *P. inui*, another species of simian malaria parasite that has a high possibility of becoming zoonotic. Although there has not yet been a report of naturally acquired *P. inui* in humans, experimentally it has been shown that humans are susceptible to this species and will develop fever if infected [70]. Another simian *Plasmodium* that is of great concern is *P. cynomolgi* as this has been reported infecting humans under natural conditions [71, 72]. Furthermore, the real number of *P. cynomolgi* infection in humans might be underestimated as this species could be misidentified as *P. vivax* by microscopy [71].

Although there was no difference in vector densities, molecular results indicated that a higher proportion (65.2%) of infected *An. balabacensis* was caught earlier in the evening between 18:00 h and midnight. Although these numbers are limited, this suggests people may be at a higher risk of getting infected with malaria during this period, especially when they are still outside their houses.

## Conclusions

The dominant *Anopheles* species in Kudat district is *An. balabacensis* which could be found equally abundant in different types of habitats, suggesting people are at a risk of exposure to infected vectors across a range of environments. Further work is needed to characterise the breeding sites within these different habitats. While limited numbers of infected *An. balabacensis* were caught, the presence of infected vectors in oil palm estates demonstrates the need for vector control measures targeting these habitats. Furthermore, mixed infections with *P. knowlesi* and other zoonotic malaria species illustrates the potential for spillover into human populations and the need for further surveillance. While deforestation and agricultural expansion can create economic opportunities, these changes may also increase spatial overlap between human, macaque and vector populations in new habitat types, presenting a challenge for malaria elimination.

## Additional files

**Additional file 1: Table S1.** PCR primers used for detecting *Plasmodium* spp. in *Anopheles* specimens.

**Additional file 2: Figure S1.** Amplification of known *Plasmodium* DNA using *Plasmodium* genus and species-specific PCR primers. Lanes M: 1.0 kb DNA ladder (Promega); Lane a: genus *Plasmodium*, 240 bp (rPLU3 + rPLU4); Lane b: *P. coatneyi*, 504 bp (PctF1 + PctR1); Lane c: *P. inui*, 479 bp (PinF2 + INAR3); Lane d: *P. fieldi*, 421 bp (PfldF1 + PfldR2); Lane e: *P. cynomolgi*, 137 bp (CY2F + CY4R); Lane f: *P. knowlesi*, 424 bp (PkF1140 + PkR1550); Lane g: *P. falciparum*, 370 bp (NewPLFshort + FARshort); Lane h: *P. vivax*, 476 bp (NewPLFshort + VIRshort); Lane i: *P. malariae*, 241 bp (NewPLFshort + MARshort); Lane j: *P. ovale*, 407 bp (NewPLFshort + OVRshort); Lane k: negative control (no DNA template).

**Additional file 3: Table S2.** Mean interaction statistic between habitat type and time period as predicted by GLMM. Habitat types: FE: forest edge; PG: playground; LH: longhouse; OP: oil palm plantation; BU: shrub bushes. Time periods are: early night (18:00–21:00 h); late night (21:00–00:00 h); predawn (00:00–03:00 h); dawn (03:00–06:00 h).

**Additional file 4: Figure S2.** Detection and identification of *Plasmodium* in *Anopheles*. Top: *Anopheles* specimens PD171-PD183 using *Plasmodium* genus-specific PCR primers. Internal control targeting *cox*2 gene of *Anopheles* was used. The numbers represent replicate 1 and 2. Lanes M: 1.0 kb DNA ladder; J: infected *Anopheles* with *Plasmodium*; K: non-infected *Anopheles*; L: negative control (no DNA template). Bottom: *Anopheles* specimen PD178 using nine species-specific PCR primers. Lanes M: 1.0 kb DNA ladder; a: genus *Plasmodium*; b: *P. coatneyi*; c: *P. inui*; d: *P. fieldi*; e: *P. cynomolgi*; f: *P. knowlesi*; g: *P. falciparum*; h: *P. vivax*; i: *P. malariae*; j: *P. ovale*; k: negative control.

**Additional file 5: Table S3.** Total *Anopheles* specimens collected at study sites in Paus, Ranau and Keritan Ulu, Keningau.

## Data Availability

The datasets supporting the conclusions of this article are included within the article and its additional files.

## Abbreviations

PCR: polymerase chain reaction
GLMM: generalised linear mixed model
MHa: million hectares
HLC: human landing catch
DNA: deoxyribonucleic acid
DTAB: dodecyltrimethylammonium bromide
CTAB: cetyltrimethylammonium bromide
*SSU* rRNA: small subunit ribosomal RNA
*cox*2: cytochrome c oxidase subunit II
