# Supplementary material for: Effect of different habitat types on abundance and biting times of Anopheles balabacensis Baisas (Diptera: Culicidae) in Kudat district of Sabah, Malaysia
Source: Parasit Vectors. 2019 Jul 25;12:364. doi: 10.1186/s13071-019-3627-0 (PMC6659233; doi:10.1186/s13071-019-3627-0)
Supplement: Supplementary file 1 — Additional file 1: Table S1. PCR primers used for detecting Plasmodium spp. in Anopheles specimens. [file 13071_2019_3627_MOESM1_ESM.docx]

**Additional file 1: Table S1.**  PCR primers used for detecting *Plasmodium* spp. in *Anopheles* specimens.

| Target | Genus/species | Primer set for PCR | Set primer | Primer name | Sequence (5’ - 3’) | Annealing temperature (^o^C) | Size of PCR product (bp) |
| --- | --- | --- | --- | --- | --- | --- | --- |
| SSU-rRNA | *Plasmodium* genus | Sets 1 + 2 | Set 1 | rPLU1^*^ | TCAAAGATTAAGCCATGCAAGTGA | 55 | 1640 |
|  |  |  |  | rPLU5^*^ | CCTGTTGTTGCCTTAAACTCC |  |  |
|  |  |  | Set 2 | rPLU3^*^ | TTTTTATAAGGATAACTACGGAAAAGCTGT | 62 | 240 |
|  |  |  |  | rPLU4^*^ | TACCCGTCATAGCCATGTTAGGCCAATACC |  |  |
| COII | *Anopheles* genus | Sets 3 + 4 | Set 3 | COIIF^Δ^ | TCTAATATGGCAGATTAGTGCA | 55 | 791 |
|  |  |  |  | CX2R^˄^ | TGATTTAAGAGATCATTACTTGC |  |  |
|  |  |  | Set 4 | CX2F^˄^ | GGCAGATTAGTGCAATGAATT | 55 | 766 |
|  |  |  |  | COIIR^Δ^ | ACTTGCTTTCAGTCATCTAATG |  |  |
| SSU-rRNA | *P. coatneyi* | Sets 1 + 5 | Set 5 | PctF1^+^ | CGCTTTTAGCTTAAATCCACATAACAGAC | 62 | 504 |
|  |  |  |  | PctR1^+^ | GAGTCCTAACCCCGAAGGGAAAGG |  |  |
|  | *P. inui* | Sets 1 + 6 | Set 6 | PinF2^+^ | CGTATCGACTTTGTGGCATTTTTCTAC | 60 | 479 |
|  |  |  |  | INAR3^+^ | GCAATCTAAGAGTTTTAACTCCTC |  |  |
|  | *P. fieldi* | Sets 1 + 7 | Set 7 | PfldF1^+^ | GGTCTTTTTTTTGCTTCGGTAATTA | 66 | 421 |
|  |  |  |  | PfldR2^+^ | AGGCACTGAAGGAAGCAATCTAAGAGTTTC |  |  |
|  | *P. cynomolgi* | Sets 1 + 8 | Set 8 | CY2F^+^ | GATTTGCTAAATTGCGGTCG | 60 | 137 |
|  |  |  |  | CY4R^+^ | CGGTATGATAAGCCAGGGAAGT |  |  |
|  | *P. knowlesi* | Sets 1 + 9 | Set 9 | PkF1140^#^ | GATTCATCTATTAAAAATTTGCTTC | 50 | 424 |
|  |  |  |  | PkR1550^#^ | GAGTTCTAATCTCCGGAGAGAAAAGA |  |  |
|  | *P. falciparum* | Sets 1 + 10 | Set 10 | NewPLFshort^$^ | CTATCAGCTTTTGATGTTAG | 53 | 370 |
|  |  |  |  | FARshort^$^ | GTTCCCCTAGAATAGTTACA |  |  |
|  | *P. vivax* | Sets 1 + 11 | Set 11 | NewPLFshort^$^ | CTATCAGCTTTTGATGTTAG | 53 | 476 |
|  |  |  |  | VIRshort^$^ | AAGGACTTCCAAGCC |  |  |
|  | *P. malariae* | Sets 1 + 12 | Set 12 | NewPLFshort^$^ | CTATCAGCTTTTGATGTTAG | 53 | 241 |
|  |  |  |  | MARshort^$^ | TCCAATTGCCTTCTG |  |  |
|  |  |  |  |  |  |  |  |
|  | *P. ovale* | Sets 1 + 13 | Set 13 | NewPLFshort^$^ | CTATCAGCTTTTGATGTTAG | 53 | 407 |
|  |  |  |  | OVRshort^$^ | AGGAATGCAAAGARCAG |  |  |

**References**

^˄^Hawkes F, Manin BO, Ng SH, Torr SJ, Drakeley C, Chua TH, et al. Evaluation of electric nets as means to sample mosquito vectors host seeking on humans and primates. Parasit Vectors. 2017;10:338.

#Imwong M, Tanomsing N, Pukrittayakamee S, Day NPJ, White NJ, Snounou G. Spurious amplification of a *Plasmodium vivax* small-subunit RNA gene by use of primers currently used to detect *P. knowlesi*. J Clin Microbiol. 2009;47:4173-5.

^+^Lee K, Divis PCS, Zakaria SK, Matusop A, Julin RA, Conway DJ, et al. *Plasmodium knowlesi*: reservoir hosts and tracking the emergence in humans and macaques. PLoS Pathog. 2011;7:e1002015.

*Singh B, Bobogare A, Cox-Singh J, Snounou G, Abdullah MS, Rahman HA. A genus- and species-specific nested polymerase chain reaction malaria detection assay for epidemiologic studies. Am J Trop Med Hyg. 1999;60:687-92.

^$^Ta TH, Hisam S, Lanza M, Jiram AI, Ismail N, Rubio JM. First case of naturally acquired human infection with *Plasmodium cynomolgi*. Malar J. 2014;13:68.

^Δ^Yang M, Ma Y, Wu J. Mitochondrial genetic differentiation across populations of the malaria vector *Anopheles lesteri* from China (Diptera: Culicidae). Malar J. 2011;10:216.
