# Supplementary figures and images for: Effect of different habitat types on abundance and biting times of Anopheles balabacensis Baisas (Diptera: Culicidae) in Kudat district of Sabah, Malaysia
Source: Parasit Vectors. 2019 Jul 25;12:364. doi: 10.1186/s13071-019-3627-0 (PMC6659233; doi:10.1186/s13071-019-3627-0)

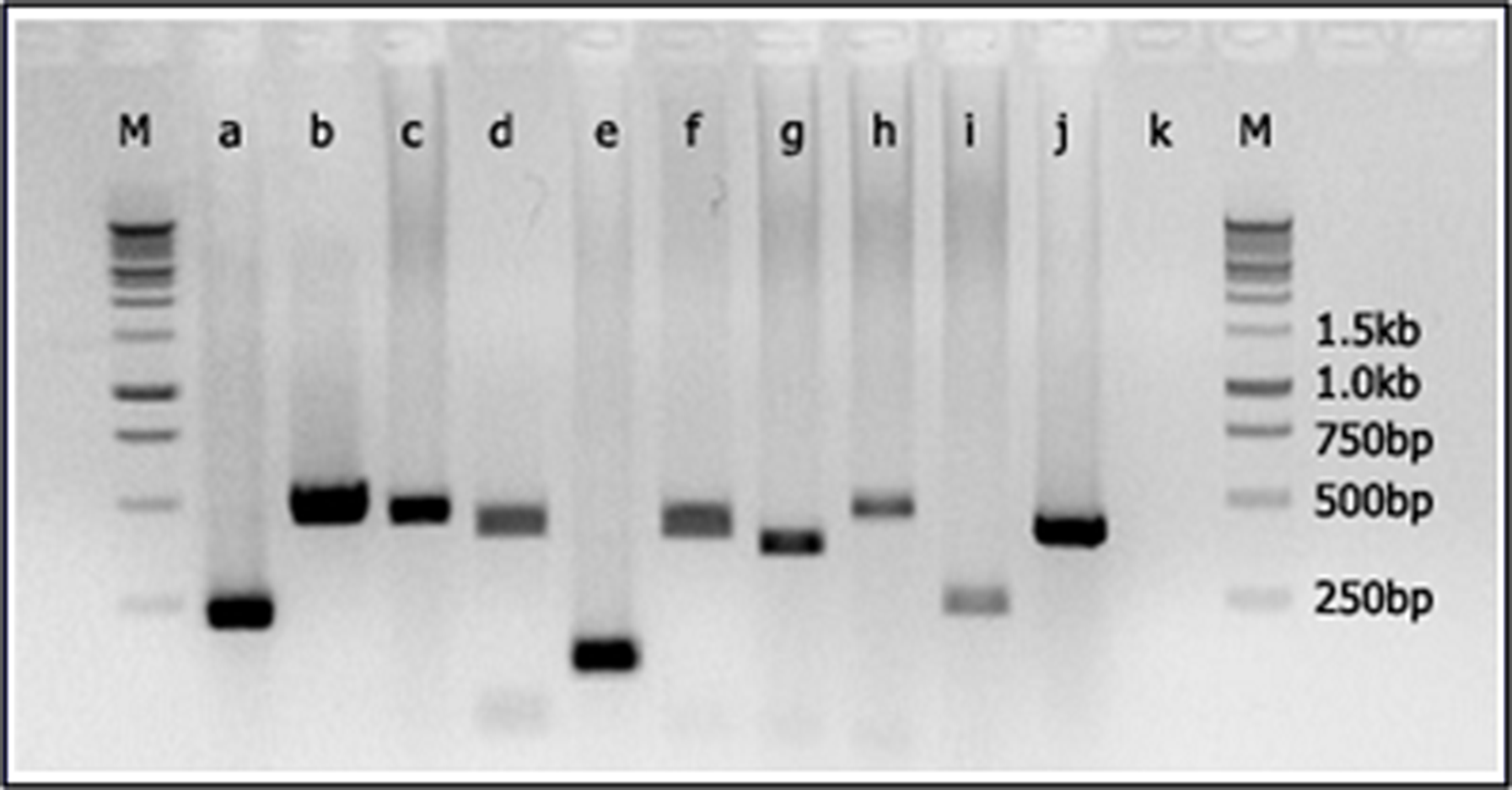

Supplement: Supplementary file 2 — Additional file 2: Figure S1. Amplification of known Plasmodium DNA using Plasmodium genus and species-specific PCR primers. Lanes M: 1.0 kb DNA ladder (Promega); Lane a: genus Plasmodium, 240 bp (rPLU3 + rPLU4); Lane b: P. coatneyi, 504 bp (PctF1 + PctR1); Lane c: P. inui, 479 bp (PinF2 + INAR3); Lane d: P. fieldi, 421 bp (PfldF1 + PfldR2); Lane e: P. cynomolgi, 137 bp (CY2F + CY4R); Lane f: P. knowlesi, 424 bp (PkF1140 + PkR1550); Lane g: P. falciparum, 370 bp (NewPLFshort + FARshort); Lane h: P. vivax, 476 bp (NewPLFshort + VIRshort); Lane i: P. malariae, 241 bp (NewPLFshort + MARshort); Lane j: P. ovale, 407 bp (NewPLFshort + OVRshort); Lane k: negative control (no DNA template). [file 13071_2019_3627_MOESM2_ESM.tif]

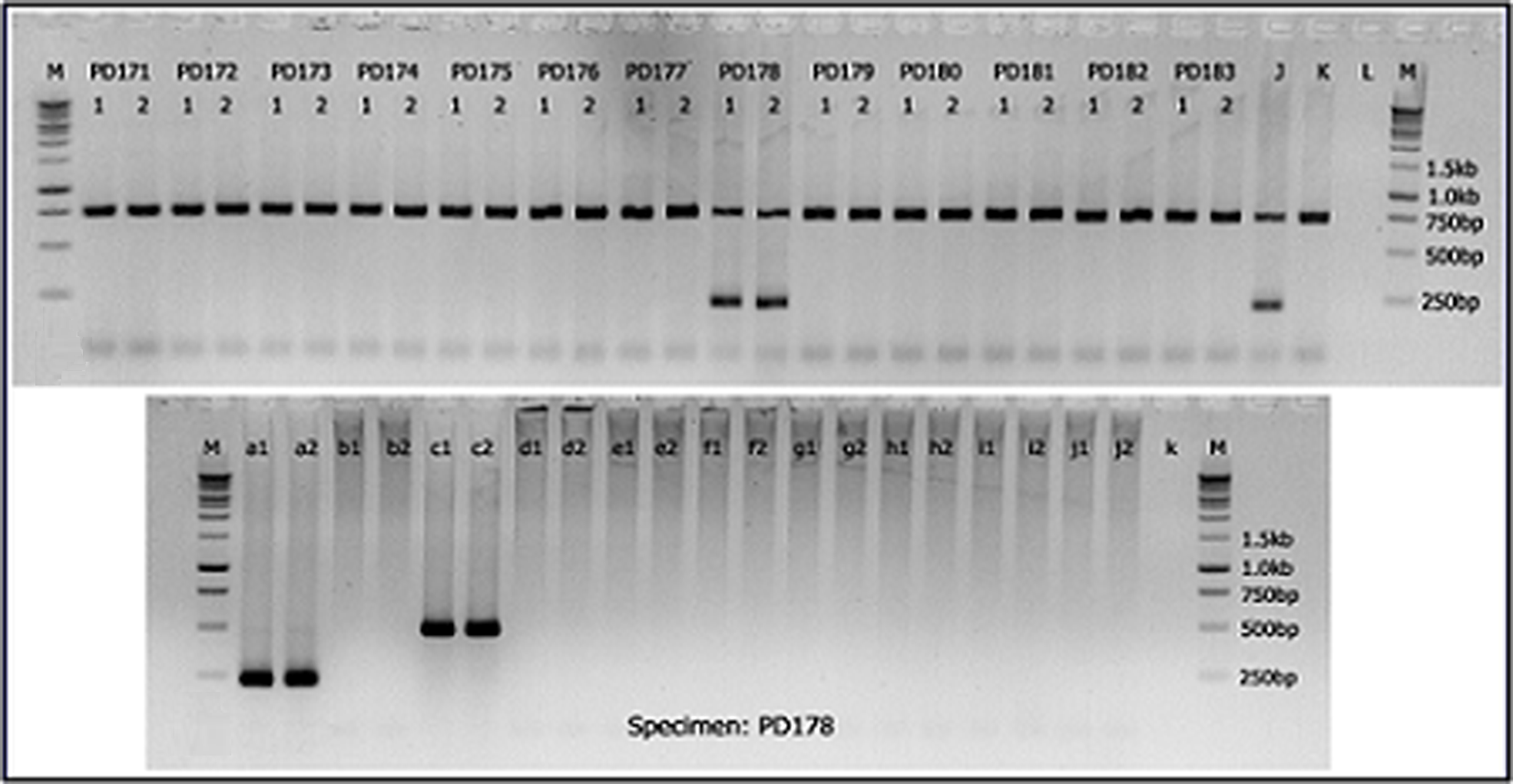

Supplement: Supplementary file 4 — Additional file 4: Figure S2. Detection and identification of Plasmodium in Anopheles. Top: Anopheles specimens PD171-PD183 using Plasmodium genus-specific PCR primers. Internal control targeting cox2 gene of Anopheles was used. The numbers represent replicate 1 and 2. Lanes M: 1.0 kb DNA ladder; J: infected Anopheles with Plasmodium; K: non-infected Anopheles; L: negative control (no DNA template). Bottom: Anopheles specimen PD178 using nine species-specific PCR primers. Lanes M: 1.0 kb DNA ladder; a: genus Plasmodium; b: P. coatneyi; c: P. inui; d: P. fieldi; e: P. cynomolgi; f: P. knowlesi; g: P. falciparum; h: P. vivax; i: P. malariae; j: P. ovale; k: negative control. [file 13071_2019_3627_MOESM4_ESM.tif]
