# Supplementary material for: Effect of different habitat types on abundance and biting times of Anopheles balabacensis Baisas (Diptera: Culicidae) in Kudat district of Sabah, Malaysia
Source: Parasit Vectors. 2019 Jul 25;12:364. doi: 10.1186/s13071-019-3627-0 (PMC6659233; doi:10.1186/s13071-019-3627-0)
Supplement: Supplementary file 3 — Additional file 3: Table S2. Mean interaction statistic between habitat type and time period as predicted by GLMM. Habitat types: FE: forest edge; PG: playground; LH: longhouse; OP: oil palm plantation; BU: shrub bushes. Time periods are: early night (18:00–21:00 h); late night (21:00–00:00 h); predawn (00:00–03:00 h); dawn (03:00–06:00 h). [file 13071_2019_3627_MOESM3_ESM.docx]

**Additional file 3: Table S2.** Mean interaction value between habitat type and time period as predicted by GLMM. Habitat types are FE: forest edge; PG: playground; LH: longhouse; OP: oil palm plantation; BU: shrub bushes. Time periods are: early night (18:00–21:00 h); late night (21:00–00:00 h); predawn (00:00–03:00 h); dawn (03:00–06:00 h).

| Interaction between habitat and time period | Mean ± SE (bites/man/time period) | Significant Tukey’s test between predicted mean |
| --- | --- | --- |
| FE*Early night | 4.7 ± 2.74 | FE*Early night - FE*Dawn: p=0.0293; |
| FE*Late night | 3.1 ± 1.90 | BU*Early night - BU*Dawn: p=0.0002; |
| FE*Predawn | 1.6 ± 1.13 | BU*Late night - BU*Dawn: p=0.0212 |
| FE*Dawn | 0.5 ± 0.28 |  |
| PG*Early night | 3.4 ± 2.05 |  |
| PG*Late night | 2.8 ± 1.68 |  |
| PG*Predawn | 1.0 ± 0.78 |  |
| PG*Dawn | 0.9 ± 0.46 |  |
| LH*Early night | 3.9 ± 2.33 |  |
| LH*Late night | 2.6 ± 1.67 |  |
| LH*Predawn | 1.2 ± 0.96 |  |
| LH*Dawn | 0.7 ± 0.37 |  |
| OP*Early night | 3.7 ± 2.25 |  |
| OP*Late night | 2.8 ± 1.74 |  |
| OP*Predawn | 1.9 ± 1.28 |  |
| OP*Dawn | 0.8 ± 0.42 |  |
| BU*Early night | 3.8 ± 1.47 |  |
| BU*Late night | 2.8 ± 1.09 |  |
| BU*Predawn | 1.2 ± 0.56 |  |
| BU*Dawn | 0.8 ± 0.24 |  |
