# Supplementary material for: Effect of different habitat types on abundance and biting times of Anopheles balabacensis Baisas (Diptera: Culicidae) in Kudat district of Sabah, Malaysia
Source: Parasit Vectors. 2019 Jul 25;12:364. doi: 10.1186/s13071-019-3627-0 (PMC6659233; doi:10.1186/s13071-019-3627-0)
Supplement: Supplementary file 5 — Additional file 5: Table S3. Total Anopheles specimens collected at study sites in Paus, Ranau and Keritan Ulu, Keningau. [file 13071_2019_3627_MOESM5_ESM.docx]

**Additional file 5: Table S3** Total *Anopheles* specimens sampled at study sites in Paus, Ranau and Keritan Ulu, Keningau.

| *Anopheles* species | Paus | | Keritan Ulu | |
| --- | --- | --- | --- | --- |
|  | Total | % | Total | % |
| *An. argyropus* | 0 | 0.0 | 1 | 0.2 |
| *An. balabacensis* | 153 | 24.7 | 102 | 22.6 |
| *An. barbumbrosus* | 120 | 19.4 | 79 | 17.5 |
| *An. donaldi* | 251 | 40.5 | 104 | 23.1 |
| *An. vagus* | 0 | 0.0 | 1 | 0.2 |
| *An. kochi* | 13 | 2.1 | 2 | 0.4 |
| *An. latens* | 1 | 0.2 | 0 | 0.0 |
| *An. maculatus* | 15 | 2.4 | 118 | 26.2 |
| *An. montanus* | 1 | 0.2 | 0 | 0.0 |
| *An. peditaeniatus* | 1 | 0.2 | 0 | 0.0 |
| *An. pujutensis* | 1 | 0.2 | 0 | 0.0 |
| *An. sundaicus* | 0 | 0.0 | 3 | 0.7 |
| *An. tessellatus* | 63 | 10.2 | 40 | 8.9 |
| *An. umbrosus* | 1 | 0.2 | 1 | 0.2 |
| Total individuals | 620 |  | 451 |  |
| Total species | 11 |  | 10 |  |
